# Glycosaminoglycans as Multifunctional Anti-Elastase and Anti-Inflammatory Drugs in Cystic Fibrosis Lung Disease

**DOI:** 10.3389/fphar.2020.01011

**Published:** 2020-07-08

**Authors:** Judith A. Voynow, Shuo Zheng, Apparao B. Kummarapurugu

**Affiliations:** Department of Pediatric Pulmonology, Children’s Hospital of Richmond at VCU, Richmond, VA, United States

**Keywords:** neutrophil elastase, cystic fibrosis, glycosaminoglycans, heparin, hyaluronic acid, High Mobility Group Box 1

## Abstract

Neutrophil elastase (NE) is a major protease in the airways of patients with cystic fibrosis (CF) that activates airway inflammation by several mechanisms. NE stimulates epithelial toll like receptors (TLR) resulting in cytokine upregulation and release, upregulates MUC5AC, a major airway mucin, degrades both phagocytic receptors and opsonins resulting in both neutrophil and macrophage phagocytic failure, generates oxidative stress *via* extracellular generation and uptake of heme free iron, and activates other proteases. Altogether, these mechanisms create a significant inflammatory challenge that impairs innate immune function and results in airway remodeling. Currently, a major gap in our therapeutic approach to CF lung disease is the lack of an effective therapeutic strategy targeting active NE and its downstream pro-inflammatory sequelae. Polysulfated glycosaminoglycans (GAGs) are potent anti-elastase drugs that have additional anti-inflammatory properties. Heparin is a prototype of a glycosaminoglycan with both anti-elastase and anti-inflammatory properties. Heparin inhibits NE in an allosteric manner with high potency. Heparin also inhibits cathepsin G, blocks P-selectin and L-selectin, hinders ligand binding to the receptor for advanced glycation endproducts, and impedes histone acetyltransferase activity which dampens cytokine transcription and High Mobility Group Box 1 release. Furthermore, nebulized heparin treatment improves outcomes for patients with chronic obstructive pulmonary disease (COPD), asthma, acute lung injury and smoke inhalation. However, the anticoagulant activity of heparin is a potential contraindication for this therapy to be developed for CF lung disease. Therefore, modified heparins and other GAGs are being developed that retain the anti-elastase and anti-inflammatory qualities of heparin with minimal to no anticoagulant activity. The modified heparin, 2-O, 3-O desulfated heparin (ODSH), maintains anti-elastase and anti-inflammatory activities *in vitro* and *in vivo*, and has little residual anticoagulant activity. Heparan sulfate with O-sulfate residues but not N-sulfate residues blocks allergic asthmatic inflammation in a murine model. Polysulfated hyaluronic acid abrogates allergen- triggered rhinosinusitis in a murine model. Finally, nonsaccharide glycosaminoglycan mimetics with specific sulfate modifications can be designed to inhibit NE activity. Altogether, these novel GAGs or GAG mimetics hold significant promise to address the unmet need for inhaled anti-elastase and anti-inflammatory therapy for patients with CF.

## Introduction

Cystic fibrosis (CF) lung disease is marked by recurrent exacerbations of acute bronchitis with an overexuberant inflammatory response and markedly high airway concentrations of neutrophil elastase (NE). A major gap in current therapy for patients with CF is the lack of anti-protease and anti-inflammatory therapies to inhibit NE and NE-activated sequelae. In this review, we will discuss the impact of NE on CF lung biology, review the current landscape of anti-protease and anti-inflammatory therapies for CF lung disease, and then discuss the biology and pharmacology of glycosaminoglycans (GAGs) as potential anti-protease and anti-inflammatory therapies for CF.

### Neutrophil Elastase and Cystic Fibrosis Lung Disease

The primary defect in CF, an autosomal recessive disorder, is the loss of function of the Cystic Fibrosis Transmembrane Conductance Regulator protein, which results in abnormal airway mucus (Stoltz et al., 2015; Boucher, 2019). CF airway mucus is tethered to submucosal ducts and airway epithelia (Ostedgaard et al., 2017; Ermund et al., 2018) with subsequent mucus stasis and failure to clear infections. Thus, recurrent cycles of infection and inflammation are established and neutrophils are recruited to the airway. In addition, mucus stasis alone may be sufficient to increase neutrophilic inflammation (Rosen et al., 2018), possibly by generating airway hypoxemic stress which triggers IL-1β and IL-1α cytokine release (Chen et al., 2019). The CF airway milieu, characterized by viscous sputum containing microbes and pro-inflammatory cytokines, further impairs neutrophil function and clearance (Voynow et al., 2008). Ultimately, in the CF airways, neutrophils release extracellular traps (Gray et al., 2018) or undergo necrosis (Vandivier et al., 2002), and release DNA and granule contents including proteases. The most abundant protease released into the CF airway is neutrophil elastase (NE).

NE is present in the bronchoalveolar lavage (BAL) fluid in infants with CF, and BAL NE concentrations are directly associated with lung disease progression starting in infancy (Sagel et al., 2012; Sly et al., 2013; Rosenow et al., 2019). NE accelerates the progression of CF lung disease by several mechanisms (Voynow et al., 2008; McKelvey et al., 2019). First, NE contributes to altered ion flux in the CF airway by activating the epithelial sodium channel (Caldwell et al., 2005) and degrading CFTR *via* an endogenous proteinase, calpain (Le Gars et al., 2013). These NE actions further aggravate altered ion and water flux across the CF airway. Second, NE activates signaling pathways that promote abnormal epithelial structure and repair. NE upregulates mucin expression and goblet cell metaplasia (Voynow et al., 2004; Park et al., 2013); and triggers epithelial apoptosis (Suzuki et al., 2009) and/or premature senescence (Fischer et al., 2013), which impair epithelial proliferation and restoration following injury. Third, NE employs several mechanisms to promote airway inflammation (Voynow et al., 2008; Bruscia and Bonfield, 2016; Roesch et al., 2018). NE amplifies inflammation by upregulating neutrophil chemokines, e.g. IL-8 (Cosgrove et al., 2011), proteolytically activating chemokines such as IL-1α or IL-33 (Clancy et al., 2018), and releasing damage associated molecular pattern proteins such as High Mobility Group Box 1 (HMGB1) (Griffin et al., 2014) which binds to the Receptor for Advanced Glycation End-products (RAGE) or facilitates ligand binding to TLR2 and TLR4 (Lotze and Tracey, 2005). NE further contributes to airway inflammation by increasing the expression of pro-inflammatory long chain ceramides (Karandashova et al., 2018; Horati et al., 2020); these lipids impact plasma membrane structure and receptor clustering. NE degrades innate immune proteins including lactoferrin and surfactant proteins A and D, and cleaves both complement and complement receptors causing impaired neutrophil and macrophage phagocytic activity (Voynow et al., 2008). NE increases the activity of other proteases; NE activates matrix metalloproteinase 9 (MMP 9) by cleavage of its prodomain and by degradation of its inhibitor, Tissue inhibitor of metalloprotease-1 (Jackson et al., 2010). In addition, the protease load is further exaggerated by the loss of endogenous anti-proteases. Anti-NE capacity is depleted in the CF airway due to NE degradation of elafin (Guyot et al., 2008), secretory leucoprotease inhibitor (Weldon et al., 2009; Twigg et al., 2015) and both oxidation and protease degradation of alpha-1- protease inhibitor (A1-PI) (Twigg et al., 2015). Finally, NE generates oxidative stress in epithelial cells and macrophages by degrading heme-containing proteins and releasing heme-free iron which is taken up by cells (Fischer et al., 2009); this process occurs in the airways of patients with CF (Ghio et al., 2013) and with COPD (Fischer et al., 2009). NE has a broad repertoire of activities that increase inflammation, impair host immunity and result in airway remodeling. Although NE appears to be a central regulator of inflammation in CF lung disease, NE actions are amplified by ligand-receptor interactions, oxidative stress, and the presence of other active proteases that contribute to a complex pro-inflammatory milieu. This may be one reason why the strategy of therapy for a single target, NE activity, in the CF airway, has not yet been successful.

### Status of Current Anti-Proteases and Anti-Inflammatory Therapies for Cystic Fibrosis

Currently, there are two anti-inflammatory therapies approved for CF: azithromycin for patients with *Pseudomonas aeruginosa* infections (Nichols et al., 2020) and ibuprofen high dose oral therapy (Konstan et al., 1995). These therapies blunt the rate of decline of lung function over time; however, they do not resolve the high airway protease load that is associated with progression of bronchiectasis and lung injury. Many anti-protease candidate drugs have been tested in the CF airway (reviewed in (Voynow et al., 2008) and (Twigg et al., 2015)). An oral neutrophil elastase inhibitor, AZD9668, was tested in a Phase II randomized, double-blind, placebo-controlled trial in patients with CF (Elborn et al., 2012). Although AZD9668 treatment was associated with decreased urine desmosine, a marker of NE activity, and decreased sputum IL-6 and Regulated on Activation, Normal T Expressed and Secreted (RANTES), there was no improvement in sputum NE activity, sputum neutrophil counts, or measures of quality of life. A recent Phase IIa randomized, placebo-controlled clinical trial of inhaled alpha1 proteinase inhibitor (A1-HC) (Gaggar et al., 2016) revealed that the treatment group had increased sputum concentrations of A1-HC, but there was no significant change in lung function, quality of life measures, or sputum NE activity or sputum cytokine levels. Recently, an inhaled anti-NE therapy, POL6014, was studied in a Phase I trial using an ascending dose schedule in both healthy volunteers and participants with CF (Barth et al., 2019). A single inhaled dose was safe in both healthy volunteers and subjects with CF. Sputum active NE levels were reduced by greater than 1-log at 3 h after treatment at all doses. Therapy with POL6014 for subjects with CF is currently being evaluated in a Phase IIa/IIb randomized, placebo-controlled, double-blind study (NCT03748199). This initial report of POL6014 activity is promising; however, there is still a compelling need to develop drugs with multifunctional anti-protease and anti-inflammatory activities that are resistant to protease degradation or oxidation.

### GAGs: Structure and Function

**Figure 1:**
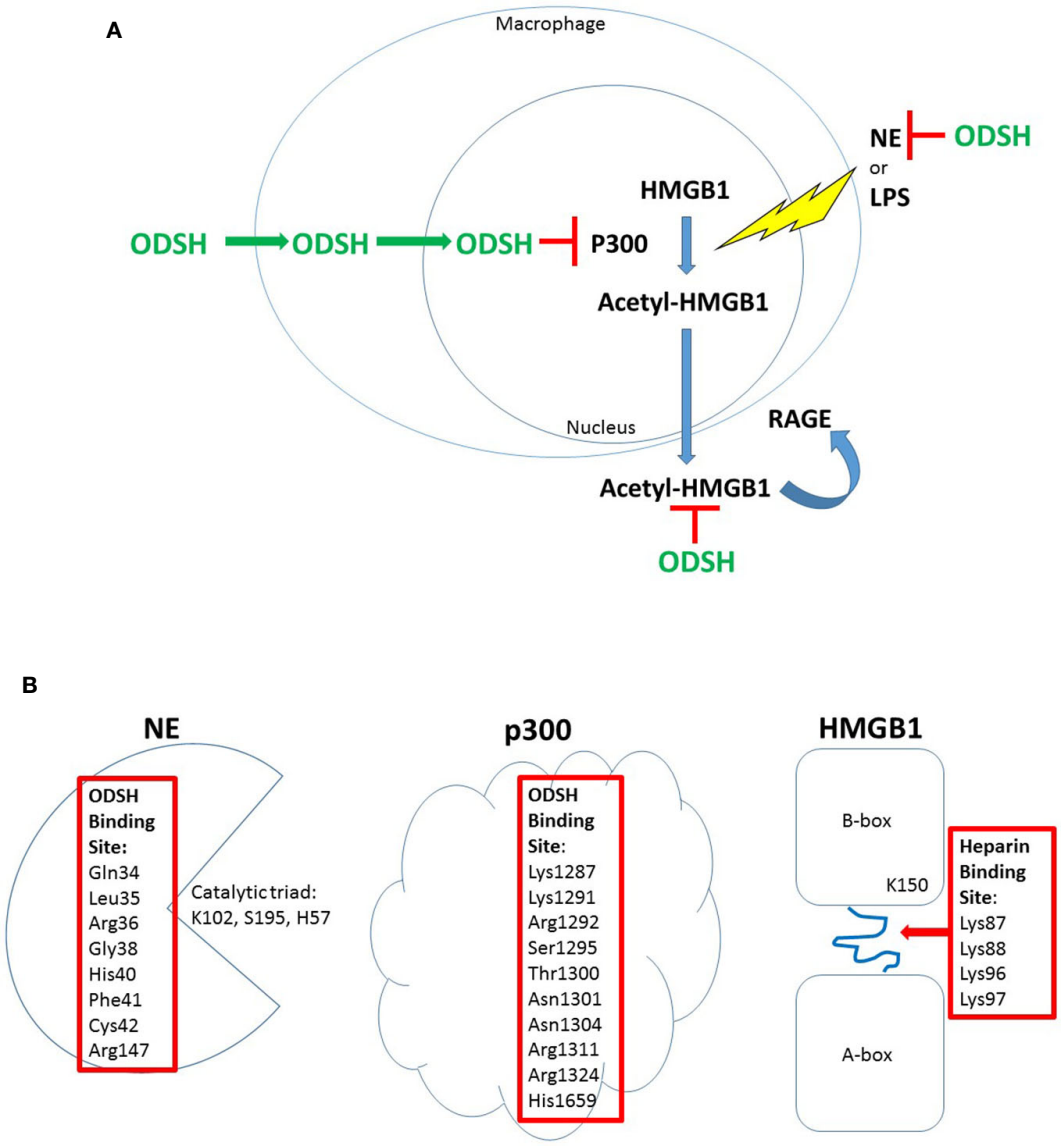
ODSH localization and function in a macrophage cell line. ODSH is taken up by a mouse macrophage cell line (RAW264.7) into the cytoplasm within 2 h and into the nucleus by 24 h (Zheng et al., 2017) **(A)**. ODSH has anti-NE activity and blocks HMGB1-RAGE interaction in the extracellular domain, and inhibits p300 lysine acetyltransferase activity in the nucleus **(A)**. ODSH inhibits NE activity by binding to an allosteric inhibitory site (Kummarapurugu et al., 2018), and ODSH inhibits p300 enzyme activity by binding to the acetyl-CoA binding site in the catalytic domain (Zheng et al., 2017) **(B)**. In contrast, ODSH binds to the loop connecting the A-box and B-box of HMGB1, blocking interaction with heparan sulfate proteoglycans required for HMGB1 ligation of the RAGE receptor (Xu et al., 2011) **(B)**. Amino acid residues required for ODSH or heparin inhibitory activity are shown (Red Box).

GAGs are polymers composed primarily of disaccharides which consist of a D-glucosamine bound to either uronic acid (D-glucuronic acid or L-iduronic acid) or galactose (Morla, 2019). The composition and linkage of monosaccharides and addition of modifications define the four major classes of GAGs: heparin/heparan sulfate (HS), chondroitin sulfate, dermatan sulfate, and hyaluronan. The uronic acid has a carboxylic acid unit and both monosaccharides are decorated with N- and O-linked sulfate residues that together confer a negative charge to the polymers. Native hyaluronan is not sulfated. In the CF lung, there are high levels of chondroitin sulfate and hyaluronan. Chondroitin sulfate proteoglycans contribute to turbidity and the mass of insoluble pellet in CF sputum; these qualities are relieved by depolymerization with chondroitinase ABC (Khatri et al., 2003). Low molecular weight hyaluronan may contribute to inflammation in the CF lung *via* TLR2 and TLR4 signaling and downstream NK-κB signaling (reviewed in (Reeves et al., 2011)). However, GAG structures can be modified to alter sulfation which plays a critical role in mediating biological effects. Moreover, GAG mimetics are being generated to achieve optimal drug characteristics while minimizing adverse properties. Heparin and HS proteoglycans bind to predicted basic amino acid-rich domains (Cardin and Weintraub, 1989; Hileman et al., 1998). GAGs have many biological effects that impact coagulation, infection, inflammation, cell adhesion, metastasis, cell matrix structure, and tissue differentiation and repair (Lima et al., 2017; Morla, 2019). Importantly, heparin can be taken up by cells and localized to cytoplasm and nucleus (Richardson et al., 2001; Raman et al., 2013) **(****Figure 1A****)**. The localization of administered heparin to both extracellular and intracellular domains permits a wide array of anticipated functions including enzyme inhibition and interference with cell- cell receptor interactions, microbe- cell interactions, and HS proteoglycan pro-inflammatory activities. In this review, we will focus on GAG properties that impact CF and other chronic lung diseases.

### Modified Non-Anticoagulant Heparins and Anti-Inflammatory Activity

Heparin is well known for its anticoagulant activity, but in addition, heparin has a broad repertoire of anti-inflammatory functions including anti-NE and anti-cathepsin G activity, inhibition of NF-kB, blockade of L- and P-selectin binding, and interference with HMGB1 release and interaction with its receptor, RAGE (Morla, 2019; Mulloy, 2019). At least three modified heparins have been developed to reduce anti-coagulant activity but retain anti-inflammatory activity: glycol-split heparin, sulfated-non-anticoagulant Low Molecular Weight Heparin (S- NACH) and 2-O, 3-O desulfated heparin (ODSH). Glycol-split heparin, generated by periodate oxidation of porcine mucosal heparin, is characterized by a cleavage between C2 and C3 of the nonsulfated uronic acid residue (Naggi et al., 2005). Glycol split heparin, administered subcutaneously to mice daily starting 10 days after establishment of chronic *P. aeruginosa-*agar bead pneumonia, decreases inflammatory cytokines, BAL neutrophil counts, and bacterial lung burden at 28 days (Lore et al., 2018). S-NACH is a purified fraction of low molecular weight heparin isolated to select drug with minimal anti-coagulant activity (Shastri et al., 2015). In a murine asthma model generated by ovalbumin (OVA)-sensitization and challenge, S-NACH intraperitoneal administration following OVA challenge blunted BAL inflammation by eosinophils, macrophages, and neutrophils, blocked goblet cell metaplasia, and blocked T2 cytokine expression in serum and BAL (Ghonim et al., 2018).

Of the modified non-anticoagulant heparins tested for anti-inflammatory efficacy, there is the most experience with 2-O, 3-O, desulfated heparin (ODSH). Fryer et al. (1997) lyophilized heparin under alkaline condition to produce ODSH. ODSH has substantially reduced anticoagulant activity compared to heparin as determined by activated partial thromboplastin time (APTT) and anti-Xa clotting assays. But the anti-neutrophil protease activities, including anti-NE and anti-cathepsin G activities, are largely unchanged in ODSH compared to heparin. ODSH also retains the pharmacological properties of heparin *in vivo*, including inhibition of bronchial hyperreactivity after antigen challenge, and prevention of airway smooth muscle cell proliferation (Fryer et al., 1997). Importantly, ODSH does not bind to platelet factor 4 and thus doesn’t trigger heparin-induced thrombocytopenia (Rao et al., 2010). ODSH interrupts ligand-receptor interactions, blunting pro-inflammatory signaling cascades. Both heparin and ODSH inhibit RAGE- HMGB1 interaction and RAGE-S100A9/calgranulin interaction *in vitro* (Rao et al., 2010). The ODSH concentrations (IC_50_) to inhibit NE activity (0.14 μg/ml), and to block HMGB1-RAGE binding (0.23 μg/ml) are similar, supporting the concept that ODSH (MW approximately 10 kD) achieves both anti-protease and anti-inflammatory activities within a nanomolar concentration range.

### Heparin and ODSH Anti-HMGB1 Activity

HMGB1 is recognized as a major inflammatory mediator in CF plasma and sputum that is strongly associated with lung disease progression (Liou et al., 2012; Chirico et al., 2015). Therefore, HMGB1 is likely to be an important target for CF anti-inflammatory therapy. HMGB1 has two major functions; it is a nuclear non-histone chromatin binding protein that facilitates transcriptional regulation, and it is an extracellular damage associated molecule pattern or alarmin that is secreted by activated macrophages as a delayed mediator of inflammation (Lotze and Tracey, 2005). HMGB1 release is triggered by HMGB1 lysine acetylation which is activated following exposure to microbial products (LPS), cytokines (TNFα) (Lotze and Tracey, 2005), or NE (Griffin et al., 2014). HMGB1 can also be released from necrotic cells. HMGB1 has been reported to transduce cellular signals by interacting with at least three receptors: RAGE, TLR2 and TLR4 (Park et al., 2004; Sharma et al., 2014). Binding of HMGB1 to RAGE activates NF-κB and the ERK/p38 pathway which promotes cytokine production (TNF, IL-6, and IFN-γ). Binding of HMGB1 to TLR2/TLR4 leads to NF-κB activation through a MyD88 (myeloid differentiation primary-response protein 88)-dependent mechanism. Importantly, ODSH blocks both HMGB1 release and HMGB1 ligation of receptors both *in vitro* and *in vivo*. Intratracheal HMGB1 in a mouse model induces significant pulmonary inflammation with increased BAL total cells, neutrophils, and TNF-α levels at 24 hr. Simultaneous intratracheal ODSH administered with HMGB1 decreased all of these BAL measures, indicating that ODSH can inhibit HMGB1-RAGE- induced inflammatory responses *in vivo* (Rao et al., 2010). A summary of glycol split heparin, S-NACH, and ODSH activities *in vivo* in preclinical models relevant to CF is summarized in **Table 1**.

**Table 1:** *In vivo* models of chronic lung diseases treated with modified or non-saccharide GAGs.

Animal Model	Treatment (Dose and Administration)	Outcome Measures	Reference
Balb/c mice: NE airway inflammation model NE (o.a.) ± **ODSH** (o.a.)	Days 1, 4, 7: NE (44 μM) or NS **ODSH** (635 μM) or NS o.a. Day 8: BAL/lung harvest	NE induces BAL cells & PMN, KC, HMGB1 **ODSH**+NE: decreases total cells and PMN; decreases KC and HMGB1	Griffin et al. (2014)
C57BL/6 mice: *P. aeruginosa* (PA01) pneumonia model PA01 (i.n.) PA01 (i.t.) ± **ODSH** (s.c.)	Day 1: PA01 i.n. **ODSH** (8.3- 75 mg/kg) or NS s.c. q 12 h x 2 Day 2: BAL/lung harvest Day 1: PA01 i.t. **ODSH** (75 mg/kg) or NS s.c. 12 h x 4 Day 3: survival	**ODSH** decreases PA01 CFU; decreases lung protein content and edema; decreases total and PMN cell count; decreases BAL HMGB1; inhibits TLR2 and TLR4 binding **ODSH** improves mouse survival	Sharma et al. (2014)
C57Bl/6N *P.aeruginosa* pneumonia model (PA) CF isolate AA43- embedded in agar beads (i.t.) ± **glycol split LMWH, C3gs20 vs. N-acetyl LMWH, C23** s.c.	Day 1: PA- agar beads (1-2 x 10^6^) vs. sterile beads i.t. Day 1-14: **C3gs20 or C23** (30 mg/kg/d) or vehicle s.c. Day 14: BAL and lung harvest Day 1: PA- agar beads (1-2 x 10^6^) vs. sterile beads i.t. Day 10-28: **C3gs20 or C23** (30 mg/kg/d) or vehicle s.c. Day 28: BAL and lung harvest	**C23** decreased BAL total cells and PMN; No significant change in PA CFU. **C3gs and C23** decreased BAL total cells and PMN, decreased total PA CFU, and decreased IL-17A **C3gs20** decreased IL-1β, IL-12pp40, G-CSF, and KC	Lore et al. (2018)
C57BL/6J mice Allergic Asthma model OVA i.p. sensitization and challenge with Ova ± **sulfated non-anticoagulant LMWH (S-NACH) i.p**.	Wk 1: Alum/Ova i.p.once per wk x 2 Wk 2-4: Ova 3% inhaled 3x per week **S-NACH** (10 mg/kg) or NS i.p. Week 5: BAL and lung harvest	**S-NACH** decreased Ova-triggered eosinophils, macrophages, lymphocytes in BAL, decreased goblet cell metaplasia, decreased lung tissue hydroxyproline, decreased BAL and serum T2 cytokines, decreased Ova-IgE.	Ghonim et al. (2018)
C57BL/6 mice: LL-37- induced rhinosinusitis model LL37 i.n.± **polysulfated HA (GM-0111)** or HA i.n.	Day 1: LL-37 (115 μg) **GM-0111** or HA (800μg) Day 2: sinus harvest	LL-37 increases Mast cells, MPO, lamina propria (LP) thickening and cell death **GM-0111**+LL-37: Decreased Mast cells, MPO, LP thickening and cell death **GM-0111** more effective than HA	Pulsipher et al. (2017)
BALB/c mice: Aspergillus chronic rhinosinusitis (CRS) model A.fumigatus extract ± **polysulfated HA (GM-1111) or PBS** i.n. 3 Groups: 1. 1. PBS 2. 2. A fumigatus+ PBS 3. 3. A. fumigatus+ GM-1111	Week 0: All groups sensitized with Alum + PBS or *A.fumigatus* i.p. Weeks 1-8: PBS or *A.fumigatus* extract (20,000 PNU i.n.) 3 x per wk. Weeks 5-8: PBS or GM-1111 (600 μg) i.n.5x per wk Week 9: Collect blood and sinonasal tissue	**GM-1111***+ A.fumigatus* (Af) extract decreased Af-induced CRS symptoms, mucosal edema and injury, goblet cells, TLR2 and TLR4, T2 cytokines, and IgE	Alt et al. (2018)
C57BL/6 mice Second hand smoke model of lung disease ± **sulfated semisynthetic HA GAG ethers (SAGEs)**	SHS vs. Rm air nasal inhalation 10 min/day x 5 d/wk 4 weeks exposure **SAGE** (30 mg/kg) i.p. for 3 d/wk Collect BAL and lung RNA and protein	**SAGEs** effect on SHS exposure: Blocked lung RAGE expression Blocked BAL protein, total cells, and cytokines: IL-α, IL-2, TNFα	Tsai et al. (2019)
Sprague Dawley rats Rat Emphysema Model with SU51416 (VEGFR inhibitor)± **polysulfated dehydropolymer of caffeic acid (CDSO3)** 3 Groups: Untreated healthy SU5416 + NS SU5416 + **CDSO3**	Day 1: SU5416 (20 mg/kg) s.c. ± Day 1–Day 21: **CDSO3** (60 μg/kg) or NS inhaled 3x per week	**CDSO3** prevented SU5416-induced emphysema, improved rat exercise endurance, decreased oxidative stress, and increased VEGF and HIF-1α, and decreased cleaved caspase-3	Truong et al. (2017)

BAL, bronchoalveolar lavage; HIF-1α, hypoxia inducible factor- 1α; i.n., intranasal; i.p., intraperitoneal; i.t., intratracheal; KC, keratinocyte chemoattractant; LMWH, low molecular weight heparin; MPO, myeloperoxidase; PMN, neutrophil; PNU, protein neoantigen units; o.a., oropharyngeal aspiration; RAGE, receptor for advanced glycation end products; s.c., subcutaneous; SHS, second hand smoke; TNFα, tumor necrosis factor α; VEGF, vascular endothelial growth factor.

The bolded text are to emphasize the stimuli for the model type and the drugs used to treat this model.

ODSH is effective in preclinical models of infection and inflammation to blunt these pathologic processes. In a *P. aeruginosa* (PA)-induced murine pneumonia model, intranasal ODSH decreases BAL HMGB1 levels, reduces pulmonary bacterial burden, ameliorates PA-induced lung injury, and improves survival (Sharma et al., 2014). In a murine model of intratracheal NE-induced lung inflammation and remodeling, ODSH pretreatment blocks NE-induced neutrophil influx, upregulation of KC, and release of HMGB1 into BAL (Griffin et al., 2014). To investigate the mechanism of ODSH inhibition of HMGB1 release, the impact of fluorescein-labeled (FITC)-ODSH on NE- or LPS-treated mouse macrophage cells (RAW264.7) was investigated. ODSH is taken up by RAW264.7 cells, and is localized to the cytoplasm and nucleus (Zheng et al., 2017). The sulfation pattern of modified heparins influence intracellular uptake and localization that is specific for different cell types (Raman et al., 2013). In RAW264.7 cells treated with NE or LPS, ODSH blocks HMGB1 lysine-acetylation in a dose-dependent manner, by inhibiting P300 histone (lysine) acetyltransferase (HAT) activity. Spectrofluorometry reveals that ODSH binding to p300 results in a conformational change in p300, and further tightens ODSH-p300 binding; this mechanism is supported by a complementary approach of in silico modeling with combinatorial virtual library screening of interactions between p300 and ODSH (Zheng et al., 2017) (**Figure 1B**). Importantly, heparin also interacts directly with HMGB1, changing its conformation and reducing its affinity for RAGE which interrupts the HMGB1-RAGE signaling cascade (Ling et al., 2011). Furthermore, heparin and ODSH bind to NE and inhibit its activity.

### Heparin and ODSH Anti-NE Activity in *Ex Vivo* CF Sputum

High concentrations of NE released by neutrophils are found in CF sputum. Importantly NE, a cationic serine protease, binds to the copious polyanionic polymers in sputum including DNA (Gray et al., 2015); mucins (Nadziejko and Finkelstein, 1994) and actin filaments (Broughton-Head et al., 2007; Kater et al., 2007). Dornase alfa (Fuchs et al., 1994) and 7% hypertonic saline (HTS) (Elkins et al., 2006), the mainstay mucoactive therapies for patients with CF, improve pulmonary function, and decrease the frequency of pulmonary exacerbations. However, both therapies have been reported to increase NE activity in CF sputum (Cantin, 1998; Chen et al., 2006). ODSH is a robust inhibitor of NE activity *in vitro* with a low IC_50_ (Griffin et al., 2014; Kummarapurugu et al., 2018), but in CF sputum, both ODSH and heparin inhibition of NE activity requires DNA depolymerization by DNase-1 (Kummarapurugu et al., 2018). This observation suggests that anionic DNA polymers compete with anionic ODSH for binding to NE. When these interactions were investigated, it was discovered by both pharmacokinetic studies and by combinatorial virtual library screening, that both DNA and ODSH bind to the same allosteric domain on NE that is required for inhibition (Kummarapurugu et al., 2018) (**Figure 1B**). Furthermore, inhibition of NE activity in sputum by heparin or DNA is chain length dependent, with a requirement for a larger size than approximately 15 monosaccharides for heparins (Spencer et al., 2006; Kummarapurugu et al., 2018) or 12-mer for DNA oligomers (Kummarapurugu et al., 2018). Neither fondiparinux, a heparin pentasaccharide (1.8 kDa) nor a DNA hexamer have anti-NE activity, confirming that a threshold length is necessary for heparin and DNA to bind to NE and exert anti-elastase activity (Kummarapurugu et al., 2018). Interestingly, unfractionated heparin releases soluble DNA from sputum that is available for dornase alfa cleavage (Broughton-Head et al., 2007). Thus, heparin enhances DNase activity.

### Novel Glycosaminoglycan Therapeutics as Anti-Protease, Anti-Microbial, and/or Anti-Inflammatory Therapies

Glycosaminoglycans have a broad array of functions both in native tissues and when modified to be used as competitors for endogenous heparan sulfate proteoglycans or for their properties to bind to cationic proteins and modify activities. Development of small synthetic non-saccharide glycosaminoglycan mimetics (NSGMs) offer modifiable alternatives for polysaccharide GAGs. NSGM 32 (Morla et al., 2019) has robust anti-elastase activity *in vitro* and has a mixed allosteric and orthosteric mechanism of action. However, NSGM 32 requires DNA depolymerization for anti-elastase activity in CF sputum, and is less potent than ODSH (Kummarapurugu et al., 2018). It was speculated that NSGM 32 binds to other positively charged moieties in CF sputum and therefore higher concentrations of drug are required for inhibition of NE activity (Morla et al., 2019). A sulfated synthetic lignin, sulfated dehydropolymer caffeic acid (CDSO3), inhibits the development of emphysema in a VEGFR-inhibitor-induced rat model *via* multiple functions including anti-oxidant activity, and prevention of epithelial and endothelial cell death *via* iron-chelation- induced stabilization of HIF-1α and VEGF signaling (Truong et al., 2017). These two compounds illustrate the exquisite target specificity due to sulfation patterns incorporated into small synthetically produced GAG mimetics. Another advantage of synthetic sulfated lignins is that they are homogeneous compounds that do not require porcine or bovine bioproducts for production.

Polysulfated hyaluronan is a modified hyaluronic acid which has potent anti-inflammatory properties (Zhang et al., 2011). Low molecular weight polysulfated hyaluronan blocks LPS-stimulated macrophage release of cytokines including TNFα, IL-6, IL-12, MCP-1, and increases expression of antioxidants, superoxide 2 and 3 (Jouy et al., 2017). In a murine model of second hand smoke induced lung disease, a polysulfated hyaluronan administered by intraperitoneal (i.p.) injection inhibits release of BAL TNFα, IL-1α, and IL-2, and decreases BAL inflammation and lung permeability (Tsai et al., 2019). A sulfated semisynthetic low molecular weight hyaluronan, GM-1111, (molecular weight 5.5 kD), has been tested for anti-inflammatory properties. In a mouse model of rhinosinusitis generated by intranasal administration of a cathelicidin fragment, LL37, GM-1111 blocks neutrophil and mast cell mucosal infiltration and significantly decreases epithelial apoptosis (Pulsipher et al., 2017). In vitro, in nasal epithelial cells, LL37 stimulates inflammation and cell death; another GM compound, GM-0111, inhibits IL-6 and IL-8 release and blocks Caspase-1- and Caspase-8 -induced cell death (Thomas et al., 2017). In an *A.fumigatus*- intranasal allergen-sensitization mouse model of chronic rhinosinusitis, intranasal GM-1111 introduced 3 weeks after *A. fumigatus* sensitization, significantly inhibits goblet cell metaplasia and mucosal T2 inflammation, and decreases TLR2 and TLR4 expression (Alt et al., 2018). In addition, in a periodontitis model, GM-0111 suppresses the growth of *P. gingivalis* and *A. actinomycetemcomitans* and biofilm formation, demonstrating antimicrobial activity (Savage et al., 2016). A summary of polysulfated hyaluronan activities *in vivo* in preclinical models of chronic lung disease is summarized in **Table 1**.

**Figure 2:**
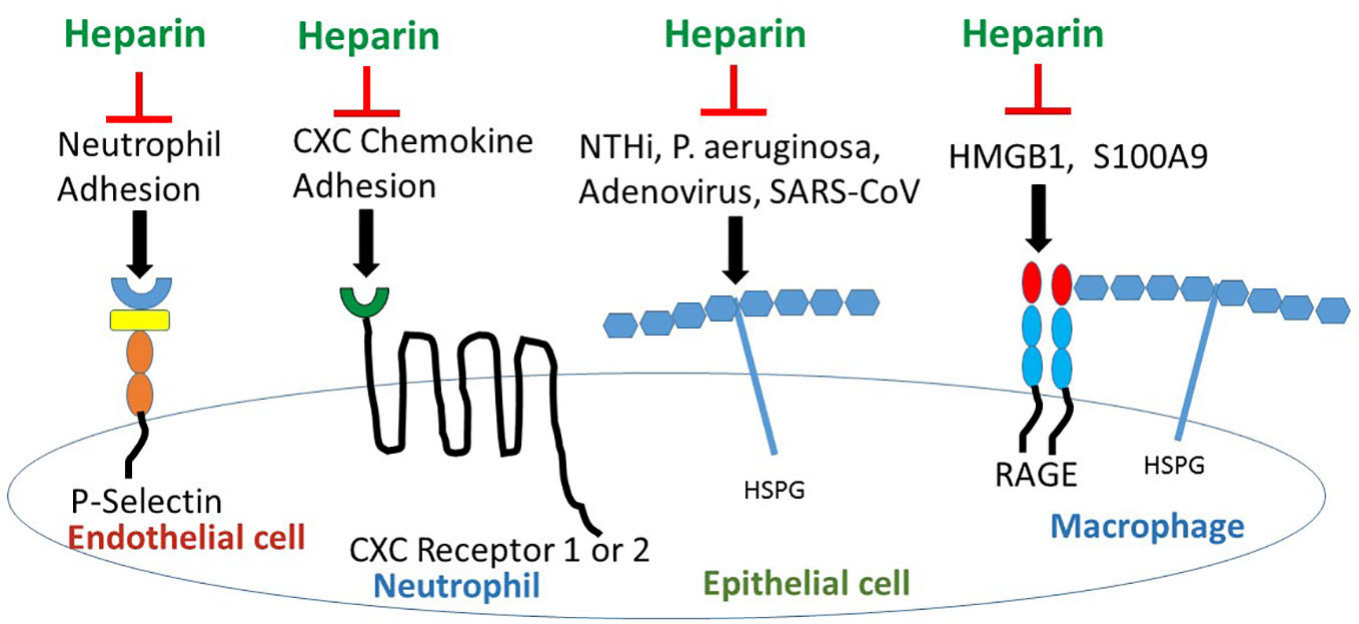
Heparin/ODSH interrupts cell- cell interactions and ligand-receptor binding to block pro-inflammatory pathways. Heparin/ODSH oligosaccharides bind to P- and L-selectins and block neutrophil adhesion and chemotaxis (Nelson et al., 1993; Rao et al., 2010). Heparin inhibits CXCL8/IL-8 and other ELR (Glu Leu Arg)-CXC chemokines from binding to G-protein coupled receptors CXCR1 and CXCR2 (Rajarathnam KaD, 2020). Heparin competes with HSPG for binding to microbial proteins which prevents bacterial or viral-epithelial adhesion and invasion (Rostand and Esko, 1997). Heparin/ODSH bind to HMGB1 and S100A9 and interrupt RAGE ligation (Rao et al., 2010). HSPG, heparan sulfate proteoglycan; NTHi, non-typeable H. influenza; S100A9, calgranulin; SARS-CoV, Severe acute respiratory syndrome- corona virus.

Heparan sulfate (HS) is expressed widely on many cell types as a proteoglycan. HS proteoglycans regulate inflammation by binding to ELR (Glu Leu Arg)- CXC chemokines at conserved His, Lys, Arg residues, controlling chemotactic gradients in the extracellular and pericellular matrices (Rajarathnam KaD, 2020). However, in the CF lung, endogenous HS proteoglycans have pro-inflammatory properties (Reeves et al., 2011); HS stabilizes cytokine and chemokine ligands, preventing protease digestion, thus increasing CXCL ligation to CXCR1 and 2 to upregulate inflammation (Rajarathnam KaD, 2020). HS enables RAGE hexamer formation for more efficient intracellular signaling (Xu et al., 2013), and binds L-selectin to promote neutrophil slowing and diapedesis across endothelial cells into tissues (Farrugia et al., 2018). HS also serves as a cell receptor for microbe adhesion and invasion (Rostand and Esko, 1997) (**Figure 2**). Bacteria, *P. aeruginosa* (Paulsson et al., 2019) and *nontypable H. influenza* (NTHi) (Su et al., 2019), and viruses, adenovirus (Dechecchi et al., 2001) and Severe Acute Respiratory Syndrome-Coronavirus (SARS-CoV) (Lang et al., 2011) and SARS-CoV-2 spike protein (So Young Kim et al., 2020) all bind to HS proteoglycans. Importantly, exposure to heparin competes with and inhibits binding to HS proteoglycans (**Figure 2**) resulting in inhibition of binding of *P. aeruginosa* (Paulsson et al., 2019) and NTHi (Su et al., 2019) to laminin, a major component of the basal lamina in the airway, and inhibition of binding of adenovirus (Dechecchi et al., 2001) and SARS-CoV (Lang et al., 2011) to epithelia, and inhibition of SARS-CoV-2 (So Young Kim et al., 2020) spike protein to HS as detected by surface plasmon resonance. Treatment with synthetic HS or heparin inhibits cytokine/chemokine binding to G-protein coupled receptors and blocks neutrophil interaction with endothelial selectins resulting in decreased neutrophil influx (Lore et al., 2018) (**Figure 2**). Altered sulfation of HS affects pro- vs anti-inflammatory behavior; increased N- and 6-O-sulfation increase cytokine ligation and neutrophil recruitment while increased 2-O-sulfation blunts neutrophilic inflammation (Axelsson et al., 2012). HS is also required for HMGB1-RAGE receptor binding; heparin can compete with HS and interrupt RAGE ligation by binding to HMGB1 (Xu et al., 2011).

### Clinical Trials Using GAGs for Respiratory Diseases

Unfractionated heparin is the only GAG used in clinical trials to date. Inhaled heparin was tested in healthy volunteers and is safe and well tolerated. When delivered by nebulization, approximately 8% of the nebulized dose of heparin is delivered to the lower respiratory tract (Bendstrup et al., 1999). Importantly, inhaled doses up to 400,000 IU, did not affect lung function, but did increase circulating anti-Factor Xa activity and activated partial thromboplastin time (APTT) (Bendstrup et al., 2002). BAL fluid was tested for anti-coagulant activity in the presence of control plasma and by this method, the half-life of inhaled heparin was determined to be 28 h (Markart et al., 2010).

Inhalation of unfractionated heparin has been tested as a therapeutic for severe COPD (Shute et al., 2018), asthma (Yildiz-Pekoz and Ozsoy, 2017), smoke inhalation (Miller et al., 2014), and acute lung injury (ALI) (Dixon et al., 2010; Tuinman et al., 2012; Juschten et al., 2017), but the number of randomized, double-blind, placebo-controlled studies for these indications is limited (**Table 2**). There is one randomized, double- blind, placebo-controlled, crossover trial of twice daily inhaled heparin (50,000 IU per dose) for 2 weeks in adults with CF, which demonstrates a good safety profile, but does not show any significant improvement in lung function, sputum inflammatory markers or mucus clearance (Serisier et al., 2006). In contrast, a randomized, double-blind, placebo-controlled single site study for COPD using twice daily inhaled heparin (150,000 IU per dose) in addition to inhaled twice daily salbutamol & beclomethasone and airway clearance for 21 days reveals that heparin improves lung function including FEV_1_, 6 minute walk distance, and Borg dyspnea score (Shute et al., 2018). The contrast between the COPD study (Shute et al., 2018) and the previously cited CF study (Serisier et al., 2006) suggests that possible reasons for the failure of heparin to improve lung function in patients with CF were an insufficient dose of heparin and/or a limited trial duration to observe clinically significant changes in pulmonary function.

**Table 2:** Clinical trials using heparin for chronic lung diseases*.

Disease	Trial design	Drug: dose and administration mode	Outcomes compared to placebo	Reference
Cystic Fibrosis	R, PC, DB- 2 weeks; CF adults; moderate to severe lung disease;N=18	Heparin (50,000 U) inhaled every 12 h	No change in FEV_1_ serum CRP sputum IL-8, MPO, NE, TCC, sputum volume	Serisier et al. (2006)
COPD	R, PC, DB- 3 weeks; COPD- GOLD II- IV; N=40	Heparin (75,000 or 150,000 IU) Inhaled twice per day	Adherence 56% Improved FEV_1_ Improved 6MWD Increased SpO2	Shute et al. (2018)
Asthma	R, PC, DB crossover; Allergic to dust mite; N=10	Heparin (20,000 U) inhaled 10 min before inhaled dust mite extract bronchoprovocation challenge	Heparin increased the Log_2_ provocation dose of dust mite protein nitrogen units causing 20% fall in FEV_1_	Bowler et al. (1993)
Asthma	R, PC, DB crossover; Allergic to dust mite; N=8	Heparin (1000 U/kg/dose) inhaled: 90 min and 30 min pre-dust mite inhaled challenge, and 2, 4, 6 h post-dust mite inhaled challenge	Heparin blunted the severity of FEV_1_% decrease in late asthmatic responses compared to placebo	Diamant et al. (1996)
Asthma EIB	R, PC, SB, cross-over—5 days; Asymptomatic; N = 12	Day 1: baseline PFT and exercise challenge; Day 3-5: Heparin (1000 U/kg) or cromolyn (20 mg) or placebo inhaled followed by exercise challenge	Heparin blocks post-exercise decrease in SGaw	Ahmed et al. (1993)
Asthma EIB	R, PC, DB, cross-over -7 days;Asymptomatic; N=13	Day 1: baseline PFT and exercise challenge; days 3–7: inhaled Heparin (80,0000 U) or Enoxaparin (0.5, 1, 2 mg/kg) or placebo 45 min before baseline PFTs and then serially post-exercise	Decrease in FEV_1_ was blocked by heparin and enoxaparin	Ahmed et al. (1999)

*Only trials with randomized, double or single blind, placebo controlled design were included. 6MWD, 6 minute walk distance test; CRP, C-reactive protein; DB, double-blind; EIB, exercise-induced bronchospasm; FEV_1_, Forced expiratory volume at 1 sec; MPO, myeloperoxidase; NE, neutrophil elastase; PC, placebo controlled; R, randomized; SB, single-blind; SGaw, Specific conductance of the airways; SpO2, oxyhemoglobin saturation; TCC, terminal complement complex.

## Summary

There are many challenges for developing anti-protease and anti-inflammatory drugs for patients with CF. The innate immune response is impaired for both viral (Zheng et al., 2003; Berkebile et al., 2020) and bacterial infections. The CF airway milieu is typified by high concentrations of several proteases including neutrophil serine proteases: NE, proteinase 3, Cathepsin G; lysosomal proteases: Cathepsins B, L, and S; and matrix metalloproteases: MMP-9, MMP-8 and MMP-12 (McKelvey et al., 2019) which stimulate downstream signaling cascades that perpetuate oxidative stress and inflammation. The strategy of directing therapy to one target is unlikely to be successful to control inflammation and prevent lung injury. Instead, we propose that GAGs can be developed and harnessed as multi-functional anti-elastase and anti-inflammatory therapies and serve an important function as part of the armamentarium for CF lung disease.

## Author Contributions

All authors wrote the text and edited the text. JV designed the figures.

## Funding

We acknowledge the following support: Department of Defense grant PR180925, Cystic Fibrosis Foundation research grants, Voynow15I0 and Voynow19G0, and Commonwealth Health Research Board grant 236-14-14 (JV).

## Conflict of Interest

The authors declare that the research was conducted in the absence of any commercial or financial relationships that could be construed as a potential conflict of interest.
